# Multidrug-Resistant Tuberculosis in HIV-Negative Patients, Buenos Aires, Argentina

**DOI:** 10.3201/eid0908.020474

**Published:** 2003-08

**Authors:** Domingo Palmero, Viviana Ritacco, Martha Ambroggi, Marcela Natiello, Lucía Barrera, Lilian Capone, Alicia Dambrosi, Martha Di Lonardo, Nélida Isola, Susana Poggi, Marisa Vescovo, Eduardo Abbate

**Affiliations:** * Hospital F. J. Muñiz, Buenos Aires, Argentina; † National Institute of Infectious Diseases “Carlos G. Malbran,” Buenos Aires, Argentina; ‡ University of Buenos Aires, Buenos Aires, Argentina

**Keywords:** tuberculosis, multidrug resistance, RFLP, HIV seronegativity, research

## Abstract

Initial multidrug-resistant (MDR) tuberculosis (TB) in HIV-negative patients treated at a Buenos Aires referral hospital from 1991 to 2000 was examined by using molecular clustering of available isolates. Of 291 HIV-negative MDRTB patients, 79 were initially MDR. We observed an ascending trend of initial MDRTB during this decade (p=0.0033). The M strain, which was responsible for an institutional AIDS-associated outbreak that peaked in 1995 to 1997, caused 24 of the 49 initial MDRTB cases available for restriction fragment length polymorphism. Of those, 21 were diagnosed in 1997 or later. Hospital exposure increased the risk of acquiring M strain–associated MDRTB by approximately two and a half times. The emergence of initial MDRTB among HIV-negative patients after 1997 was apparently a sequel of the AIDS-related outbreak. Because the prevalence of M strain–associated disease in the study population did not level out by the end of the decade, further expansion of this disease is possible.

Multidrug-resistant (MDR) tuberculosis (TB) in patients with a history of previous TB treatment is known as acquired MDRTB and usually reflects shortcomings in current treatment administration such as irregular drug supplies, inadequate treatment regimens, or poor patient compliance (*1*). On the other hand, MDR among new cases of TB (initial MDRTB) originates from infectious sources that are disseminating MDR bacilli in the community; this resistance is an indicator of treatment deficiencies accumulated over a prolonged period (*2*).

During the last decade, MDRTB has surged as an important global problem. The AIDS pandemic fostered this emergence by spreading the disease among immunodeficient institutionalized patients (*3*–*5*). According to the global antituberculosis drug resistance surveillance program of the World Heath Organization and International Union Against Tuberculosis and Lung Disease, Argentina was identified as an MDRTB “hot spot” in the mid-1990s (*6*). At that time, initial MDRTB in that country was fueled by contemporary AIDS-related hospital outbreaks, the most important of which originated at Muñiz Hospital (*7*–*12*).

Located in Buenos Aires City, Muñiz Hospital is the main Argentinean referral center for infectious diseases and provides for approximately 800 TB patients yearly. An appreciable proportion of the MDRTB cases diagnosed in the country (mainly from the Buenos Aires metropolitan area) are referred to this center for further bacteriologic assessment and clinical management. Soon after the emergence of HIV infection in 1982, TB grew into the most frequent AIDS-associated disease in the country. During the 1990s, the hospital underwent an extensive AIDS-related MDRTB outbreak while continuing to provide care for HIV-negative drug-resistant TB patients.

Throughout the 1990s, a total of 736 AIDS-related MDRTB cases were hospitalized in Muñiz Hospital (1 [1991], 4 [1992], 10 [1993], 58 [1994], 149 [1995], 132 [1996], 159 [1997], 108 [1998], 79 [1999], and 36 [2000]; unpub. data). An IS*6110* restriction fragment length polymorphism (RFLP) study conducted in the first half of the decade of a sample of AIDS-related TB cases resistant to five or more drugs identified the so-called M strain as the main outbreak strain (*9*). Our RFLP database documents the predominance of the M strain among AIDS-related MDRTB cases investigated in this hospital during the second half of the decade (*10*). The infection disseminated to other healthcare centers in the metropolitan area, and secondary microepidemics were reported to be associated to the M strain (*11*,*12*).

Beginning in 1995, Muñiz Hospital implemented a number of measures to stop the spread of MDRTB. Isolation rooms with portable HEPA filters became available in HIV buildings, and a separate ward was dedicated to HIV-associated MDRTB. Following the recommendations of a visiting team from the Division for TB Elimination at the Centers for Disease Control and Prevention, additional measures were gradually implemented beginning in 1996, including smear examination at admission, speeding of bacteriologic diagnosis through radiometric bacteriologic assay, ready availability of second-line drugs, and personal respiratory protection with N-95 masks (*13*).

Our study was directed at gaining insight into the impact of the MDRTB epidemic that occurred in Buenos Aires in the putatively immunocompetent population. We examined the trend of initial MDRTB in non-HIV patients treated at the Muñiz Hospital during the last decade in the light of molecular clustering of available isolates.

## Methods

### Population

Medical records of all HIV-negative patients with MDRTB (both inpatients and outpatients) diagnosed at Muñiz Hospital from January 1991 to December 2000 were reviewed. All patients were routinely tested for HIV infection by enzyme-linked immunosorbent assay after giving informed consent. The following patient data were collected: year of MDRTB diagnosis, age, gender, nationality, place of residence, site of TB disease, chest x-ray score of lesion extension (minimal, moderate, advanced), history of previous TB treatment, and underlying illnesses. MDRTB cases were defined as those caused by *Mycobacterium tuberculosis* resistant to at least isoniazid and rifampicin. MDRTB patients with a history of previous TB chemotherapy were defined as acquired MDRTB cases. Initial MDRTB was defined as that diagnosed in a patient without previous TB treatment. Initial MDRTB cases were classified according to possible exposure settings, including hospital (patients with a history of previous hospitalization or hospital attendance and healthcare workers), household, and undisclosed source of infection.

### Bacteriologic Methods

Susceptibility to isoniazid, rifampicin, ethambutol, and streptomycin was determined according to World Health Organization standards. Susceptibility to kanamycin, p-aminosalycilic acid, and cycloserine was performed according to the Canetti, Rist, and Grosset method, whereas the pyrazinamidase test was used to infer pyrazinamide susceptibility (*14*).

### DNA Fingerprinting

Available isolates of initial MDRTB cases were typed by IS*6110* DNA fingerprinting by using the standardized protocol for *M. tuberculosis* (*15*). Briefly, DNA was extracted from bacilli suspensions and digested with the restriction enzyme *Pvu*II. DNA fragments were electrophoresed in 0.8% agarose and vacuum-blotted into a positively charged nylon membrane. The IS*6110* probe was a 245-bp DNA fragment amplified by polymerase chain reaction and labeled by the enhanced chemiluminescence gene detection system (Amersham International plc, Amersham, U.K.). *M. tuberculosis* Mtb 14323 was used as a reference strain. Hybridization patterns were compared visually. Isolates from one patient were considered to be part of a cluster if the IS*6110* fingerprint matched that of a different patient within the study period.

### Statistics

We used the Mantel-Haenszel test to compare categoric data from groups of patients. For the comparison of age means, the Student t test was applied. The relative weight of initial MDRTB throughout the study period was analyzed by the Mantel test for linear trend. P values <0.05 were considered significant. The software used was Epi Info version 6.0 (*16*).

## Results

From January 1991 to December 2000, a total of 291 HIV-negative patients treated at Muñiz Hospital were affected by MDRTB. Of these, 212 (72.9%) were acquired MDRTB cases, and 79 (27.1%) were unambiguously identified as initially MDR. The primary site of disease was pulmonary in all patients of both groups with a single exception. An accidental subcutaneous inoculation of a bacteriologist caused the only primary extrapulmonary case, which was obviously classified in the initial MDRTB group.

**Figure 1:**
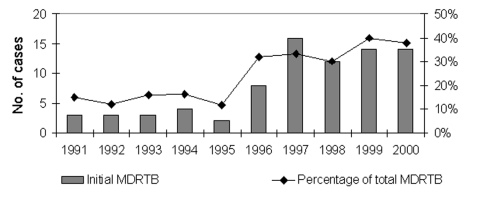
Trend of initial multidrug-resistant tuberculosis among HIV-negative patients at Muñiz Hospital, Buenos Aires, 1991–2000. MDRTB, multidrug-resistant tuberculosis.

Demographic, clinical, and bacteriologic characteristics of both groups are compared in Table 1. Compared with acquired-MDRTB patients, patients with initial MDRTB showed significant differences in age, gender, and chest x-ray involvement. The presence of an underlying illness was not identified as a predisposing factor for initial MDRTB. Initial MDRTB patients were significantly younger and their lung lesions less advanced. Female patients predominated in this group. Initial MDRTB increased from 15.0% of total MDR cases in 1991 to 37.8% in 2000 (test for trend, p=0.00033, odds ratio [OR] = 3.45) (Figure 1).

**Table 1:** Demographic, clinical, and bacteriologic features of acquired versus initial multidrug-resistant tuberculosis (MDRTB) cases among 291 HIV-negative patients, Muñiz Hospital, Argentina, 1991–2000

	Acquired MDRTB (n=212)	Initial MDRTB (n=79)	p value
Age (y ± SD)	39.8±13.2	35.3±15.0	0.013
Gender (male/female)	125/87	32/47	0.005
Origin (Argentinean/foreign-born)^a^	180/32	64/15	0.533
Chest x-ray (minimal/moderate/advanced)	2/52/158	10/38/31	<0.0001
Underlying illness (Y/N)	46/166	15/64	0.731
Diabetes (N)	26	7	–
Alcoholism (N)	9	1	–
Silicosis (N)	2	1	–
Steroid treatment (N)	2	3	–
Substance abuse (N)	1	0	–
Malignancy (N)	1	2	–
Other (N)	5	1	–
Drug resistance, median number of drugs (range)	4 (2–8)	4 (2–6)	–

^a^Neighboring countries (Peru, Bolivia, Paraguay).

**Figure 2:**
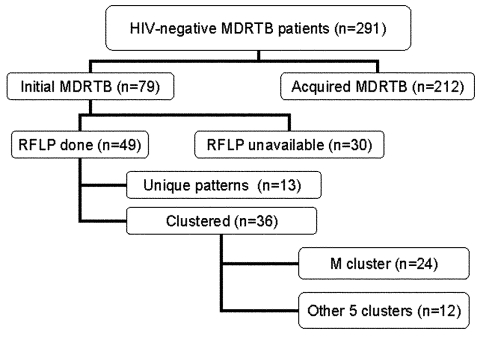
HIV-negative multidrug-resistant tuberculosis groups investigated. MDRTB, multidrug-resistant tuberculosis; RFLP, restriction fragment length polymorphism.

Isolates from 49 of the 79 patients with initial MDRTB were available for DNA typing. Thirty-six (73.0%) of these 49 fit in six molecular clusters with RFLP patterns of six or more bands. The M strain was responsible for the largest cluster involving 24 (49.0%) of the 49 investigated cases. All patients in the M cluster lived and were treated in Buenos Aires City area. A flow tree is shown in Figure 2.

The second largest cluster occurred in four members of a Peruvian family who had immigrated to Argentina in 1999. The remaining four clusters consisted of two cases each. Samples from cases in two of these clusters had matching fingerprints with previously documented outbreak strains; the patients’ histories were consistent with such hospital-acquired disease (*7*). The remaining four case-patients in the cluster were household contacts of two clearly identified index MDRTB cases.

**Figure 3:**
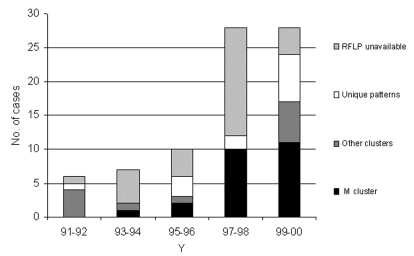
Biennial number of initial multidrug-resistant tuberculosis cases among HIV-negative patients and restriction fragment length polymorphism findings. RFLP, restriction fragment length polymorphism.

Figure 3 shows the biennial number of initial MDRTB cases attributable to the M strain, other strains, and those unavailable for RFLP. Among those available for fingerprinting, the proportion of initial MDRTB associated with the M strain increased significantly in the period 1997–2000 when compared with previous years (21/36 vs. 3/13; p=0.03; OR= 0.21).

The putative exposure setting and RFLP findings of the 79 initial MDRTB cases are described in Table 2. Hospital-related disease attributable to the M strain was detected in 15 cases from seven different health institutions in the metropolitan area. The M strain–associated disease was significantly more frequent among hospital-exposed cases when compared with patients who acquired the disease elsewhere (15/20 vs. 9/29; p=0.002; relative risk [RR] = 2.42; 95% confidence interval [CI] 1.33 to 4.40). The proportion of isolates lost for RFLP typing did not differ significantly between the groups compared above to analyze prevalence of the M strain in different periods (p=0.52) and exposure settings (p=0.11).

**Table 2:** Distribution of 79 HIV-negative patients with initial multidrug-resistant tuberculosis (MTDRTB) according to the putative exposure setting and DNA fingerprint findings, Muñiz Hospital, Argentina, 1991–2000

Setting	n (% of total)	RFLP done^a^	M strain
Hospital			
Healthcare workers^b^	20 (25.3)	15	10
Patients	7 (8.9)	5	5
Household	35 (44.3)	19	5
Unknown	17 (21.5)	10	4
Total	79 (100)	49	24

^a^RFLP, restriction fragment length polymorphism.

^b^Physicians, 6; nurses, 5; bacteriologists, 2; and auxiliary staff, 7.

## Discussion

Initial MDRTB is contracted from a source case, whereas acquired MDRTB is the result of a prolonged history of inadequate treatment. Therefore, patients with initial MDRTB were substantially younger and their lung lesions less advanced. Women predominated in the initial MDRTB group, which might reflect the conventional female role as caregiver in the local population.

The ascending trend of the initial MDRTB in HIV-negative patients may be explained as a consequence of the AIDS-associated outbreak that occurred in the hospital during the 1990s. The M strain was preeminent in this phenomenon. Initial MDRTB cases attributable to this strain appeared at the peak of the AIDS-related epidemic, persisted beyond its decline, and did not reach a neat plateau at the end of the study period. These dynamics reflect the normal latency of infection and hint that a further expansion of the strain is still possible.

Hospital exposure increased the risk of acquiring M strain–associated MDRTB by approximately two and a half times. Thus, the hospital seems still to be the most likely setting for acquiring the M strain of MDRTB. However, 4 of 10 case-patients with an unidentified source of exposure were also affected by this strain. These patients were thoroughly interrogated again, and a previous institutional or household contact was virtually discarded. The failure to trace the index cases for these patients indicates the presence in the community of unidentified infectious cases harboring this highly resistant strain.

However, the marked increase of initial MDRTB cannot be ascribed entirely to the M strain. Other indigenous outbreak strains and strains recently introduced to the country contributed to this emergence, adding complexity to the MDRTB problem in the metropolitan area. Approximately one in every five case-patients with both initial and acquired MDRTB had recently migrated from a neighboring country where TB prevalence is higher than in Argentina, which suggests the influence of regional migration on local MDRTB rates.

The outcome of this investigation cannot be generalized to scenarios different from that of a referral center for infectious diseases. Other possible limitations of our study should be considered. Bias caused by laboratory cross-contamination is improbable because two or more isolates were obtained from every patient and susceptibility patterns were consistent in all cases. Data loss was appreciable: over one third of the isolates were lost for fingerprinting because of inadequate culture maintenance. Therefore, TB transmission might have been underestimated in our population, as shown by computer simulation studies (*17*,*18*). Eventually, misclassification of cases might have occurred. Some observations induced the idea of misclassification. A sudden shift from fully susceptible TB (RFLP not available) to M strain–associated MDR disease was documented in six of the cases classified in the acquired MDRTB group. We suggest that these patients were actually reinfected with the M strain while still being treated with standard chemotherapy at the hospital. As demonstrated by van Rie et al., exogenous reinfection is not uncommon among HIV-negative patients in settings with high prevalence of TB and the distinction between initial and acquired MDRTB in such situations becomes ambiguous (*19*).

The emergence of initial MDRTB among HIV-negative persons assisted in Muñiz Hospital in the second half of the 1990s can be considered a sequel to the MDRTB epidemic that occurred among AIDS patients in Buenos Aires. The M strain has already exceeded nosocomial bounds and might be expanding in the community. The strain appears to be more prosperous than other contemporary MDR strains and is emerging as a persistent strain.
